# Interactive, Image-Based Modules as a Complement to Prosection-Based Anatomy Laboratories: Multicohort Evaluation

**DOI:** 10.2196/85028

**Published:** 2026-01-13

**Authors:** Caroline Sumner, Sami L Case, Samuel Franklin, Kristen Platt

**Affiliations:** 1 College of Medicine University of Kentucky Lexington, KY United States; 2 Department of Biomedical Sciences College of Veterinary Medicine and Biomedical Sciences Colorado State University Fort Collins, CO United States; 3 Department of Neuroscience College of Medicine University of Kentucky Lexington, KY United States

**Keywords:** digital learning, education, medical, gross anatomy, prosection, hybrid anatomy education

## Abstract

**Background:**

As medical and allied health curricula adapt to increasing time constraints, ethical considerations, and resource limitations, digital innovations are becoming vital supplements to donor-based anatomy instruction. While prior studies have examined the effectiveness of prosection versus dissection and the role of digital tools in anatomy learning, few resources align interactive digital modules directly with hands-on prosection experiences.

**Objective:**

This project addresses that gap by introducing an integrated, curriculum-aligned platform for self-guided cadaveric learning.

**Methods:**

We created Anatomy Interactives, a web-based laboratory manual structured to complement prosection laboratories for MD, DPT, and PA students. Modules were developed using iSpring Suite (iSpring Solutions Incorporated) and included interactive labeled images, donor photographs, and quiz-style self-assessments. Learners engaged with modules before, during, or after laboratory sessions. PA/DPT and MD students completed postcourse surveys evaluating module use and perceived impact. MD student examination scores from a 2023 cohort (no module access) were compared to a 2024 cohort (with access) to evaluate effectiveness.

**Results:**

A total of 147 students completed the survey (31 PA/DPT and 116 MD). The majority reported using modules for 1-2 hours per week and found them helpful for both written and laboratory examinations. MD students in the 2024 cohort performed better on all 3 examinations compared to the 2023 cohort, with 2 examination median differences reaching statistical significance (Mann-Whitney U, *P*<.001). Qualitative feedback highlighted accessibility, content reinforcement, and user engagement as key benefits.

**Conclusions:**

Interactive modules integrated with prosection laboratories enhanced learner engagement and performance. This hybrid digital-donor model shows promise for scalable, learner-centered gross anatomy education.

## Introduction

Gross anatomy remains a foundational component of health professions education, providing essential knowledge for clinical reasoning, procedural competency, and disease management [1]. However, students in prosection-based courses often struggle to transition from routine passive study to active, tactile engagement during laboratory sessions. Most existing digital resources are intended for use before or after the laboratory rather than functioning as interactive tools to engage the learner at the dissection table. As a result, students may feel disconnected from the donor during prosection, limiting the depth and integration of their learning experience.

Historically, cadaveric dissection has been regarded as the pedagogical gold standard for anatomy education [2,3]. However, contemporary anatomical curricula face mounting logistical, ethical, and financial constraints. These constraints include high costs for donor procurement and preservation, limited laboratory space and faculty, exposure to formaldehyde and other chemicals, and ethical concerns. These forces are driving the adoption of alternative teaching approaches such as prosection, peer-assisted learning, and digital platforms [4].

A growing body of literature suggests that prosection-based learning can be as effective as, and in some cases more efficient than, dissection in facilitating anatomical knowledge acquisition and clinical relevance [5-7]. A comparative assessment demonstrated that nonmedical undergraduate students reported similar academic outcomes and levels of engagement regardless of whether they were taught through dissection or prosection [3]. Likewise, in a study of veterinary students, prosections prepared by qualified staff outperformed traditional dissection in promoting both efficiency and student satisfaction [8]. Coker et al [9] report that students who learned by prosection performed better on questions specifically involving complex or deep structures, but simultaneously demonstrated higher satisfaction with and preference for a dissection-based model.

Concurrently, the integration of digital technologies into anatomy curricula is transforming how learners engage with spatial content. Radiographic imaging is a time-tested methodology that has been incorporated into undergraduate medical education with increasing frequency [10-12]. Ultrasound-based anatomy instruction has grown in popularity in recent years [13,14]. In addition, meta-analytic findings underscore the effectiveness of virtual- and augmented-reality platforms in reinforcing anatomical understanding and enhancing learner autonomy [15,16]. This shift was accelerated by the COVID-19 pandemic, during which hybrid and online formats became critical for continuity in medical education [17].

Other commercially available digital tools serve to augment the didactic anatomy learning experience. For example, the Anatomage and Sectra digital dissection technologies rely on the Visible Human Project work to generate a donor experience in physical format reminiscent of a donor table [18,19]. Use of virtual dissection as a supplement to donor dissection has been viewed favorably by students [20]. The Anatomage also offers unique advantages, such as blood flow modeling that cannot be replicated in a donor [21]. However, these devices are often proposed as an alternative to the study of the donor body and do not address the gap wherein digital media can complement the study of the prosected donor in real time. Models such as hybrid prosection-based anatomy laboratories demonstrated high student satisfaction, with learners appreciating the flexibility and clarity offered by digital supplements [7].

Several institutions have developed innovative digital platforms to complement or enhance traditional gross anatomy instruction, exemplifying how technology can be leveraged to support donor-based learning. For instance, the University of Michigan’s BlueLink program offers students an extensive library of presentation slide-like materials rich with cadaveric images, facilitating self-directed study and review [22]. These resources, which also include laboratory manuals and interactive files, are designed to provide a flexible and widely accessible digital ecosystem for anatomy education. Similarly, Texas Tech University Health Sciences Center has developed a gross anatomy program featuring a varied suite of online tools [23]. Their offerings include prelaboratory cadaveric dissection videos, practice quizzes for reinforcing knowledge of cadaveric material, and various other interactive learning modules, providing multifaceted support for students beyond traditional laboratory settings.

From a theoretical perspective, multimodal learning and constructivist principles provide a useful lens for understanding why integrated digital prosection tools may enhance anatomy learning. Multimodal learning theory posits that students build stronger and more durable knowledge when information is presented through complementary channels, such as visual, spatial, textual, and tactile, which is an especially relevant consideration in gross anatomy [24]. Constructivist frameworks further suggest that learners deepen understanding by actively engaging with material, generating connections, and iteratively refining mental models during hands-on exploration, and this approach has been shown to facilitate more meaningful learning than traditional methods in the context of human anatomy [25]. Past constructivist approaches in anatomical sciences education include techniques such as casting and creating models [26,27]. Digital resources that integrate with prosection-based laboratory work have the potential to support meaning-making in real time, helping students actively construct anatomical understanding while drawing on multiple modes of representation.

Despite the growing prevalence of prosection-based anatomy coursework and the surge of fully virtual learning models, few resources are designed specifically to bridge digital learning with hands-on prosected donor experiences in a structured, curriculum-aligned format. The Anatomy Interactives platform was developed to fill this gap, providing guided modules that integrate interactive digital content with cadaveric prosections [28]. Unlike static resources or purely virtual simulations, this tool is designed to reinforce laboratory-based learning through active engagement, self-assessment, and iterative feedback in a digital format while students are present in the gross anatomy laboratory.

This study evaluates the effectiveness of the Anatomy Interactives platform in 2 different student populations and implementation formats. This includes analyses of student engagement, self-reported preparedness, and academic performance. This work will interest medical and health professions students and educators whose classroom is the cadaveric laboratory. These learners often face challenges integrating didactic anatomical knowledge with hands-on experiences, particularly when transitioning between digital prelaboratory preparation and in-laboratory prosection-based learning. By comparing student experiences and performance across 2 cohorts, one with access to the digital modules and one without, we aim to provide evidence for the impact of integrated, digital-prosection hybrid models on anatomy education for future health professionals.

## Methods

### Ethical Considerations

All work with living subjects (ie, surveys, website traffic, and grades) was reviewed by the University of Kentucky Institutional Review Board (IRB) and determined to be exempt (IRB Protocol #94449) from human subjects research oversight. All procedures comply with the World Medical Association Declaration of Helsinki. The donor registration form of the University of Kentucky Willed Body Program includes language of consent for “education, general research, and/or to further innovative technologies.” This project used anonymous, optional surveys. No identifiable or sensitive personal data were collected, and all responses were stored without linkage to participants. Informed consent was collected via the cover page of the survey with approved study details, contact information for the University of Kentucky Office of Research Integrity, and a final question asking, “Do you consent to your anonymized survey data being used in future research publications or other research purposes?” Participants received no compensation for taking part in the survey.

### Curricular Context

We implemented this educational resource as a required component of the first-year gross anatomy courses at the University of Kentucky, serving learners in the MD, DPT, and PA programs. During the course laboratory days, the medical students rotate between physical examination with point-of-care ultrasound, prosection in the gross anatomy laboratory, and radiology-focused integrated clinical case topic sessions. This manuscript only considers the gross anatomy component of the laboratory experiences. All medical students participated in donor-based laboratory sessions using prosected donors, while PA and DPT students used an alternating dissection model (half dissected one day; the other half dissected the opposite day, with peer teaching at the end of each session—a long-used dissection model) [29]. We specifically designed the Anatomy Interactives modules to supplement these in-person experiences. Students had no prerequisite training in anatomy beyond the concurrent lectures offered within the course. Facilitators (anatomy instructors) had no specific training on the Anatomy Interactives.

### Development of Anatomy Interactives

#### Overview

We created the Anatomy Interactives website to function as a digital laboratory manual, aiming to enhance anatomical structure identification and spatial reasoning through the use of prosected donor images.

Our primary goal was to provide a guided, active learning experience that reinforced anatomical concepts outside of the traditional laboratory setting. We executed the development process in 2 distinct phases, representing the type of materials that we created.

#### Phase 1: Foundational Content Module Development

**Figure 1:**
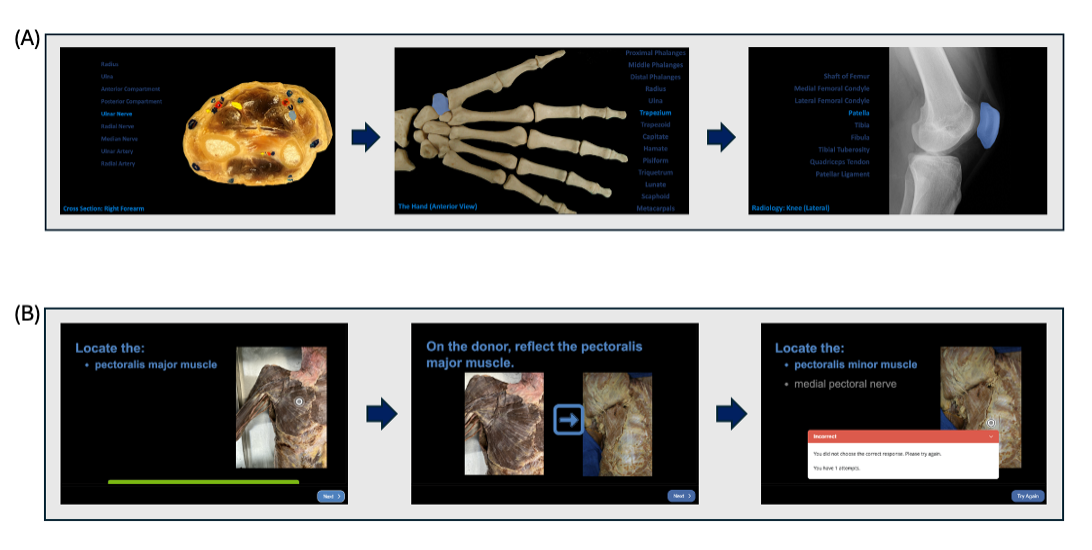
Sample view of Anatomy Interactives Website. (A) Examples of fixed labeled images from the hover-over identification resource. Screenshots from cross-sectional, osteology, and radiology image sets showing labeled anatomical structures. Structures and corresponding names are highlighted for visual reinforcement of anatomical terminology. (B) Example Anatomy Interactives module interface: pectoral muscle identification task. Screenshot from the pectoral region module showing a pin-on-image question asking students to identify a muscular structure. Immediate feedback is provided upon incorrect identification, with instructional guidance on adjusting the donor to reveal the underlying structure.

In this phase, we generated labeled anatomical images from diverse modalities, including arteriograms, cross-sections, computed tomography scans, radiographs, and osteological photographs (Figure 1A). We embedded these images with hover-over identification features, where selecting an anatomical term would highlight the corresponding structure on the image, and vice versa. We initially built this content in Microsoft PowerPoint and subsequently exported it to HTML format using iSpring Suite 11 to ensure web accessibility.

#### Phase 2: Prosection Laboratory Module Development

In the second phase, we focused on developing interactive, donor-based modules (Figure 1B). We dedicated each module to a specific anatomical subregion (eg, foot and thigh) and incorporated a series of image-based questions, such as pin-on-image tasks, multiple-choice questions, and check-all-that-apply questions. We augmented these with pop-up content designed to provide relevant clinical and conceptual context. Each module culminated in a score based on accuracy, offering students formative feedback on their understanding. We then grouped multiple modules to comprise a regional “laboratory” based on curricular structure. We developed six laboratories: (1) back and upper limb, (2) lower limb, (3) thorax, (4) abdomen, (5) pelvis, and (6) head and neck.

### Implementation

#### Overview

At the beginning of the semester, we oriented students to the Anatomy Interactives platform via an in-class demonstration. We hosted the modules on the institution’s learning management system, and they were available on demand throughout the course. We suggested students use the modules before, during, and after their scheduled laboratory sessions to reinforce learning. During the laboratory time for the MD students, the main exercise was for them to complete the module with their group (approximately 8 students per group, with 2 donors). However, we did not mandate completion of the modules for course credit for any student (MD, PA, and DPT). Students used these modules using university-sponsored iPads and any personal devices that could access the internet. The modules were all made available to students from the start of their gross anatomy course, allowing them to integrate both types of resources into prelaboratory preparation and ongoing review throughout the semester.

#### MD Students

Students in the MD program had 80-minute gross anatomy prosection laboratory sessions 6 weeks out of the 9-week course. MD students collaborated with their table group to use the digital Prosection Laboratory Modules as they worked with their donor. Students answered multiple-choice questions or select-all-that-apply questions as a team, and compared the location of the item on the hotspot questions to the anatomy of their group’s specific donor. We made the Foundational Content Modules available for students to self-study on their own time to prepare for assessments. The fall 2024 cohort of MD students had access to the Anatomy Interactives modules as part of their standard curriculum. The fall 2023 cohort of MD students, serving as a comparison group, completed the same gross anatomy course without access to these modules. Both cohorts completed the course with the same professors with nearly identical assessments. Both groups had full-time access to the anatomy laboratory and participated in the same number of small-group laboratory sessions, the only difference being the use of the modules.

#### PA/DPT students

In spring 2024, we provided PA/DPT students access during their shared dissection-based gross anatomy laboratory course. The format of this course consisted of 2 days of 2-hour lectures followed by 2-hour laboratories per week for 16 weeks. We encouraged students to use the Foundational Content Modules and Prosection Laboratory Modules as self-study to prepare for laboratories and course assessments.

### Assessment and Evaluation Strategy

#### Overview

We used a mixed-methods approach to evaluate the usage and effectiveness of the Anatomy Interactives platform. This strategy included student surveys, website usage data, and measures of academic performance.

#### Surveys

Surveys were distributed at the conclusion of the respective course to all PA/DPT students and MD students who had access to the modules. The surveys aimed to assess perceived utility, engagement, and the learning impact of the platform. Surveys included questions including which modules were used, time spent using the modules, time spent in the laboratory, opinions regarding score correlation with module usage, opinions regarding preparation level and module usage, study purposes when using the modules, and use of modules with the donor or in a stand-alone study. We collected open-ended responses pertaining to likes, dislikes, and recommendations for the modules and analyzed them thematically.

#### Website Analytics

We collected web traffic data from Google Analytics. These included metrics such as daily active users and usage patterns, particularly around examination periods and laboratory sessions, to understand engagement trends.

#### Academic Performance

We obtained written and practical anatomy examination scores from the Office of Medical Education (determined as not human research per the University of Kentucky IRB) for 2 MD student cohorts: the 2023 cohort (N=206) without access to the modules, and the 2024 cohort (N=205) with access. To ensure baseline academic comparability between student cohorts, we compared Medical College Admission Test (MCAT) scores, overall undergraduate grade point averages (uGPA), and science undergraduate grade point averages (sGPA) for both MD cohorts to test for differences. We then conducted a comparison of examination performance between these 2 MD cohorts to assess the educational impact of the Anatomy Interactives modules. We first tested for normality of the data and determined that our data was not normally distributed. Thus, we analyzed significance using an unpaired Mann-Whitney *U* test with statistical significance set at *P*<.05 and assessed effect size by calculating the rank-biserial correlation coefficient (r_rb_). Some examination scores were not available in the dataset, and therefore, the number of participants changes per examination; this was accounted for in the data analysis. Statistical analyses were conducted in GraphPad Prism (version 10.6.1; GraphPad Software, LLC). r_rb_ was manually calculated using the simplified formula as previously described [30].

## Results

Analysis of the student surveys (N=31 for PA/DPT and N=116 for MD) and website trafficking data provided significant insight into the usage of the modules by students.

**Figure 2:**
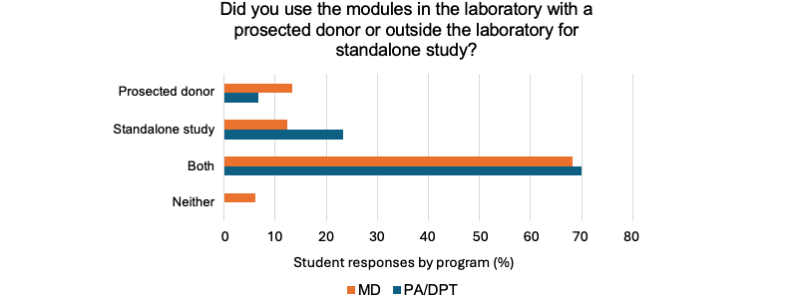
Student self-report of module usage type. Percentage of surveyed MD students (n=116) reporting use of the Anatomy Interactives modules in each context: with the donor during laboratory sessions, as a stand-alone study tool, or both. Percentage of surveyed PA/DPT students (n=31) reporting use of the modules in each context.

**Figure 3:**
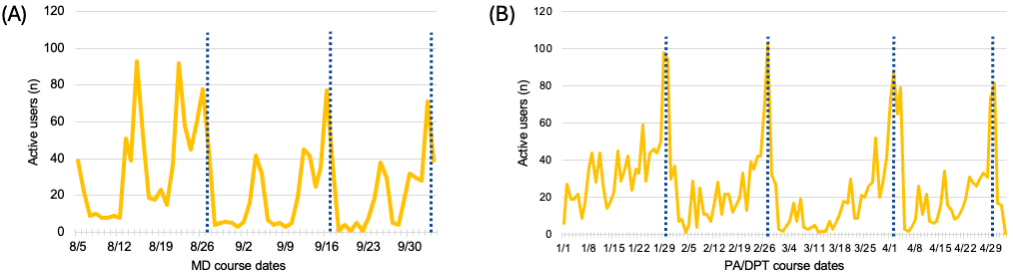
Daily users of the Anatomy Interactives platform for MD and PA/DPT courses. (A) Number of MD student users per day during the MD gross anatomy course (August 5, 2024, to October 24, 2024). (B) Number of PA/DPT student users per day during the PA/DPT gross anatomy course (January 2, 2024, to April 30, 2024). Spikes correspond to laboratory sessions and examination periods. Vertical dashed lines indicate examination dates.

For the PA/DPT cohort, 23 out of 31 (74%) students reported using the modules for 1-2 hours per week in addition to the 5-6 hours spent with the donor in allotted laboratory time. Of the medical students surveyed, 85 out of 116 (73%) students report using the laboratory modules 1-2 hours per week. Regarding the nature of their usage, 22 out of 31 (70%) PA/DPT students and 79 out of 116 (68%) medical students used the modules both with the donor and as a stand-alone study tool (Figure 2). The website data indicate the modules are heavily trafficked leading up to the examination, the number of users increasing drastically in the 1-3 days leading up to an examination for PA/DPT and medical students (Figure 2). Module page traffic also increases on laboratory days for medical students, as well as the day before and the day after a laboratory session (Figure 3). The laboratory modules had much higher traffic than the hover-over labeled images.

When asked about the relationship between their module usage and examination score, 23 out of 31 (74%) PA/DPT students reported that the modules helped them on their written examination, and 26 out of 31 (84%) believed the modules helped them on their laboratory examination. The PA/DPT cohort felt more prepared for the examination when they had a laboratory module to support the curriculum. The medical student survey revealed 80 out of 116 (69%) of that cohort believed their assessment scores reflected the time and effort they spent on modules.

To examine the modules’ impact, examination scores from 2 different cohorts were compared (Table 1). First-year medical students in 2023 did not use the modules as part of their laboratory donor experience, while the medical students in 2024 did have access to the new resource. For all 3 measures we used to test comparability (MCAT scores, uGPA, and sGPA), the data were not normally distributed (Shapiro-Wilk test, *P*<.001) and not significantly different (Mann-Whitney *U*, *P*<.001) between the 2 MD cohorts. All MD cohort examination score data failed normality testing (Shapiro-Wilk test, *P*<.001), so groups were compared using nonparametric testing. Briefly, Mann-Whitney *U* tests were used to test for cohort examination score differences, and r_rb_ values were calculated and used to assess effect size. Table 1 summarizes medians, *P* values, Mann-Whitney *U*, and the r_rb_ values from these tests. Students in the 2024 cohort scored higher on both the written and practical portions across examination 1 and examination 3, with moderate effect sizes for the written portions and total examination scores.

**Table 1:** Comparison of anatomy examination scores between MD cohorts with and without module access. Median written, laboratory, and total scores for 3 examinations in the 2023 cohort (no module use) and the 2024 cohort (module use), with *P* values, Mann-Whitney U, and rank-biserial correlations.

			2023 MD cohort, median (IQR), %	2024 MD cohort, median (IQR), %		*P* value		Mann-Whitney *U*		Rank-biserial correlation
**Examination 1 medians**										
	Written portion	82 (74-88)		86 (79-94)	<.001		15,130		.28	
	Laboratory portion	90 (85-95.5)		92.5 (85-97.5)	.01		18,153		.14	
	Total	86.1 (78.9-90)		88.9 (82.2-94.4)	<.001		15,778		.25	
**Examination 2 medians**										
	Written portion	83 (76-90)		84 (78-90)	.13		19,201		.09	
	Laboratory portion	92.5 (87.5-95)		92.5 (87.5-95)	.39		19,984		.05	
	Total	87.8 (81.1-91.1)		87.8 (82.2-92.2)	.16		19,327		.08	
**Examination 3 medians**										
	Written portion	85 (79.5-92)		92 (84-96)	<.001		12,420		.41	
	Laboratory portion	92.5 (87.5-95)		92.5 (87.5-97.5)	.49		20,092		.04	
	Total	87.8 (83.3-92.2)		92.2 (87.5-97.5)	<.001		14,645		.30	

When asked about the purpose of their module usage, 27 PA/DPT students used the modules for general studying, 8 before laboratory for preparation, 24 on laboratory day for preparation, 23 for understanding current content, and 25 for targeted examination review. A review of medical students’ qualitative feedback on the modules revealed students appreciated the accessibility of the modules, reinforcement of course content, engagement provided by the active-learning style, and the complementary nature of the modules and the prosected donor experience. Accessibility and flexibility were mentioned as advantages by 43 students, with 27 stating an appreciation for the convenience and ease of using the modules on their own time, and 16 mentioning they were helpful during independent examination preparation. The complementary nature of the modules to the prosected donor experience was highlighted by 19 students who detailed the usefulness of having the resource available during the laboratory session. Furthermore, 41 students mentioned liking how the modules support course content and reinforce lecture material. Technological characteristics were mentioned by many students, with 29 students giving positive feedback on the technology and user interface of the modules, while 48 students thought it was an area for improvement.

## Discussion

### Principal Findings

The Anatomy Interactives digital laboratory manual achieved its intended goal of supplementing the prosection-based gross anatomy curriculum with an interactive, learner-centered resource that promoted structure recognition, spatial understanding, and self-directed learning. Moving toward a student-centered learning approach in anatomy may have positive effects on student performance [31]. While learners report that student-centered approaches promote their independent learning and peer communication, they perceive that faculty-led learning increases their anatomical knowledge [32]. Before the implementation of the modules at our institution, the prosection laboratory had become a completely passive faculty-led environment with minimal student participation. By offering a digitally guided experience aligned with the prosected donor material, this innovation supported student engagement and potentially improved academic performance across multiple learner groups.

Both MD and PA/DPT learners found the modules helpful in preparing for examinations and reinforcing course content. Website analytics corroborated student usage of the modules as a study material outside of class, as website usage spiked before laboratory sessions and assessments. This suggests that the modules were integrated into learners’ study routines in a timely and meaningful way. It is still uncertain whether engagement with various digital media in anatomical education correlates to improved student outcomes and satisfaction with the learning experience [33]. However, the goal of implementing the digital modules during laboratory time was to promote engagement as a means to foster a constructivist learning environment [34]. That is to say, during the laboratory, the modules were a student-centered method to have learners interact with the donors and the course content in a meaningful way, such that they could develop or “construct” their own knowledge base as it related to the gross anatomical structures. The outcomes observed suggest that we successfully addressed the initial challenge of providing anatomy learners with a resource that bridges traditional prosection-based instruction and flexible, learner-driven study to enhance both student satisfaction and academic performance.

Most notably, medical students who used the modules in 2024 outperformed the previous cohort (2023) on both written and practical assessments, even after controlling for prior academic performance (ie, MCAT scores, uGPA, and sGPA), indicating a potential causal relationship between module usage and examination performance. While the use of MCAT scores is an imperfect means of normalizing between cohorts, it is a method that has been used by similar studies [9]. More work is warranted to assess whether the use of the digital laboratory modules directly improves course performance compared to baseline (faculty-led prosection session with no digital modules).

Recent work has drawn light to the importance of educational design that creates flourishing medical and health sciences learners and practitioners [35]. Independent of developing student knowledge recall, the anatomy laboratory offers a unique environment in which to promote the holistic development of future clinicians [36,37]. In fact, with careful course design, the anatomy laboratory can be harnessed as a means to promote the development of flourishing physicians and health care providers [38]. The Anatomy Interactives laboratory modules could be enhanced in future iterations to encourage learners to embrace a sense of meaning and belonging in line with these goals.

### Limitations

This educational resource was implemented and evaluated within a single institution, limiting the generalizability of the results. The anatomy curriculum and access to prosected donors may differ at other institutions, affecting feasibility and relevance. While the resource is available for use in any program, the module progression is modeled after the University of Kentucky’s gross anatomy curriculum and may not directly follow along the curriculum of other institutions. To access the resource, students simply need a device that connects to the internet. To implement the use of the modules in a digital-donor laboratory experience, institutions must have access to prosected donors and a space to conduct a laboratory session, likely with course director oversight.

Additionally, the evaluation relied on student self-reports and examination scores as primary metrics, which, while informative, do not fully capture long-term knowledge retention, clinical application, or behavioral change. The module usage and examination data were examined on a cohort level, rather than correlated on an individual level, which limits the interpretation of their association. Furthermore, although MCAT scores, uGPA, and sGPA were used to control for incoming academic aptitude, unmeasured variables such as instructor differences, schedule differences, or peer learning dynamics could have influenced performance outcomes.

### Future Directions

During implementation, we found that introducing the resource early in the course and orienting students to its features increased usage. Embedding the modules via a link from the learning management system allowed easy access and made integration into student study workflows more seamless. However, we also learned that variability in student technology preferences and learning styles may affect engagement; while some praised the interactivity of the modules, others noted opportunities for improvement in interface design and navigation.

Future iterations of the Anatomy Interactives platform will aim to improve user interface design, enhance accessibility across devices, and incorporate more clinically relevant content, such as radiographic correlations and pathology examples. Broader implementation at multiple institutions would help test generalizability and effectiveness across diverse learner populations and curricula. Additionally, incorporating longitudinal tracking and correlating module usage with clinical anatomy performance in later coursework or clerkships could strengthen claims about its educational impact.

On a broader scale, this work supports ongoing shifts in anatomy education toward multimodal, learner-driven resources. The findings may inform curricular policy regarding the balance between traditional laboratory experiences and flexible digital tools, particularly as programs navigate logistical constraints, student wellness, and educational equity.

## Abbreviations

IRB: Institutional Review Board
MCAT: Medical College Admission Test
sGPA: science undergraduate grade point average
uGPA: undergraduate grade point average

## Data Availability

The datasets generated or analyzed during this study are not publicly available due to concerns of student educational data protection, but are available in an anonymous format from the corresponding author on reasonable request.
